# Sensitive and fast identification of bacteria in blood samples by immunoaffinity mass spectrometry for quick BSI diagnosis†
†Electronic supplementary information (ESI) available. See DOI: 10.1039/c5sc04919a


**DOI:** 10.1039/c5sc04919a

**Published:** 2016-01-26

**Authors:** Yingdi Zhu, Liang Qiao, Michel Prudent, Alexandra Bondarenko, Natalia Gasilova, Siham Beggah Möller, Niels Lion, Horst Pick, Tianqi Gong, Zhuoxin Chen, Pengyuan Yang, Lysiane Tissières Lovey, Hubert H. Girault

**Affiliations:** a Laboratoire d'Electrochimie Physique et Analytique , École Polytechnique Fedérale de Lausanne , Rue de l'industrie 17 , CH-1951 Sion , Switzerland . Email: hubert.girault@epfl.ch; b Transfusion Interrégionale CRS , Laboratoire de Recherche sur les Produits Sanguins , CH-1015 Lausanne , Switzerland; c Department of Fundamental Microbiology , University of Lausanne , CH-1015 Lausanne , Switzerland; d Laboratoire de Chimie Physique des Polymères et Membranes , École Polytechnique Fédérale de Lausanne , CH-1015 Lausanne , Switzerland; e Institute of Biomedical Sciences , Fudan University , Dong'an Road 131 , 200032 Shanghai , China; f Hôpital du Valais, Sion , Avenue du Grand Champsec 80 , CH-1951 Sion , Switzerland

## Abstract

Bloodstream infections rank among the most serious causes of morbidity and mortality in hospitalized patients, partly due to the long period (up to one week) required for clinical diagnosis. In this work, we have developed a sensitive method to quickly and accurately identify bacteria in human blood samples by combining optimized matrix-assisted laser desorption/ionization time-of-flight mass spectrometry (MS) and efficient immunoaffinity enrichment/separation. A library of bacteria reference mass spectra at different cell numbers was firstly built. Due to a reduced sample spot size, the reference spectra could be obtained from as few as 10 to 10^2^ intact bacterial cells. Bacteria in human blood samples were then extracted using antibodies-modified magnetic beads for MS fingerprinting. By comparing the sample spectra with the reference spectra based on a cosine correlation, bacteria with concentrations as low as 500 cells per mL in blood serum and 8000 cells per mL in whole blood were identified. The proposed method was further applied to positive clinical blood cultures (BCs) provided by a local hospital, where *Escherichia coli* and *Staphylococcus aureus* were identified. Because of the method’s high sensitivity, the BC time required for diagnosis can be greatly reduced. As a proof of concept, whole blood spiked with a low initial concentration (10^2^ or 10^3^ cells per mL) of bacteria was cultured in commercial BC bottles and analysed by the developed method after different BC times. Bacteria were successfully identified after 4 hours of BC. Therefore, an entire diagnostic process could be accurately accomplished within half a day using the newly developed method, which could facilitate the timely determination of appropriate anti-bacterial therapy and decrease the risk of mortality from bloodstream infections.

## Abstract 2

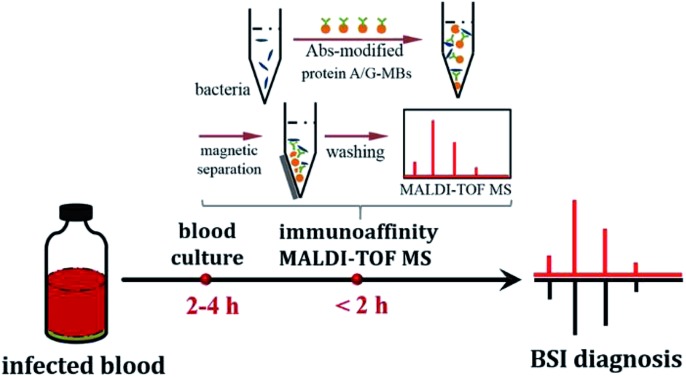
An immunoaffinity mass spectrometry method was proposed for quick BSI diagnosis.

## Introduction

Bloodstream infections (BSI), which are caused by the presence of bacteria or fungi in the bloodstream, rank among the most serious causes of morbidity and mortality in hospitalized patients.^1^ A systematic review estimated that about 600 000 BSI episodes occur in North America every year, and about 1 200 000 BSI episodes affect Europe, resulting in roughly 86 000 and 157 000 deaths, respectively.^2^ Therefore, the diagnosis and treatment of BSIs are of great importance.

Currently, blood culture (BC) methods are regarded as the “Gold Standard” for BSI diagnosis and have been widely used in clinical microbiology laboratories. In traditional BCs, large volume blood samples collected from patients (*i.e.* 20–30 mL for an adult, and 1–20 mL for a child) are injected into dedicated BC bottles and are cultured for up to 5 days or even longer (depending on the bacterial species), followed by hours to days of subculture and microorganism phenotypic identification.^3^ The whole identification process takes a relatively long time and is highly dependent on the personal experience of doctors, which forfeits BSI diagnosis at early stages of infection and increases the risk of mortality.

Rapid and accurate identification of bacteria from blood samples is crucial for effective therapy and the reduction of cost and stay-time in hospital. At present, genotypic methods, such as real-time polymerase chain reaction (PCR), fluorescence *in situ* hybridization (FISH), and 16S ribosomal RNA gene sequencing, have been developed as alternative approaches for BSI diagnosis.^4^,^5^ However, the stability and reproducibility of these methods can barely reach the requirements of clinical diagnosis. Recently, an integrated comprehensive droplet digital detection method integrating droplet microfluidics, DNAzyme-based sensors, and a high-throughput particle counter system was developed and could detect a low abundance of bacteria from *Escherichia coli*-spiked blood samples.^6^

Compared to other methods for bacteria identification, mass spectrometry provides sensitive and accurate label-free detection with high throughput. Since pioneering works in the 1990s,^7^–^9^ matrix-assisted laser desorption/ionization time-of-flight mass spectrometry (MALDI-TOF MS) has been widely used for bacteria identification at the genus, species and even strain level.^10^–^12^ With this concept, commercial MALDI-TOF MS systems, including Vitek MS (bioMérieux) and Biotyper (Bruker Daltonics), have been adopted for the identification of a variety of bacteria from blood samples, and received clearance from the US Food and Drug Administration (FDA).^13^–^15^ However, typical MALDI-TOF MS identification works only when there is a high abundance of bacteria and it is unable to identify bacteria directly from a patient’s blood, where the initial bacteria concentration is normally low, often <100 colony forming units per milliliter (CFU mL^–1^) for adult patients.^16^,^17^ Thereby, large volume blood samples and long term BCs are still needed for MALDI-TOF MS based BSI diagnosis.

Further improvements in the sensitivity of bacteria identification from blood samples by MALDI-TOF MS are crucial for fast BSI diagnosis. This can be realized by two approaches: (i) more efficient bacteria enrichment/separation, (ii) more sensitive MALDI-TOF MS detection. With respect to the first approach, affinity probes have been used for bacteria separations. For instance, Wu *et al.* used Fe_3_O_4_ NP–graphene nanosheets decorated with chitosan to capture pathogenic bacteria from aqueous suspension, and as low as 500 CFU mL^–1^*Pseudomonas aeruginosa* or 450 CFU mL^–1^*Staphylococcus aureus* in water (1 mL) were detected.^18^ Similarly, commercial anti-*Salmonella* Dynabeads® (Lake Success, NY, USA) were used to selectively isolate *Salmonella choleraesuis* for MALDI-TOF MS analysis, where the limit of detection (LOD) was demonstrated to be 10^7^ cells per mL in water, and 10^9^ cells per mL in human urine or chicken blood (1 mL).^19^ With respect to the second approach, Zenobi *et al.*^20^ recently developed functional high-density micro-arrays for the ultrasensitive analysis of single cells by MALDI-TOF MS. The micro-arrays were fabricated by laser ablation on a hydrophobic and organo-phobic layer to focus sample spots within 100 μm in diameter. In such a way, cells positions can be easily located, and a higher sample surface concentration can be achieved, leading to an enhanced signal-to-noise ratio (S/N) during analysis.

In the present work, a highly efficient immunoaffinity enrichment/separation of bacteria by antibodies-modified magnetic beads (Abs-MBs) was combined with ultrasensitive MALDI-TOF MS for bacteria identification from blood samples. A library of bacteria reference spectra was first built by collecting the MALDI-TOF MS fingerprints of different bacteria at different cell numbers. By reducing the sample spot size to 800 μm, effective reference spectra could be obtained from as few as 10 to 10^2^ bacterial cells. With this library, bacteria were identified from blood samples by spectra pattern matching. A frequently used cosine correlation method was conducted to calculate a spectral similarity score.^21^ Identification was reached based on the highest similarity score and validated with statistical confidence.

The immunoaffinity MALDI-TOF MS method was tested with three species of bacteria: *Escherichia coli* (*E. coli*), *Bacillus subtilis* (*B. subtilis*) and *Staphylococcus aureus* (*S. aureus*). Both *E. coli* and *S. aureus* are among the most common pathogens that cause BSIs worldwide.^22^,^23^ The LODs achieved were 500 cells per mL in human blood serum and 8000 cells per mL in human whole blood (1 mL) for all three bacteria. To the best of the authors' knowledge, these are the lowest reported LODs for bacteria identification from blood samples by MALDI-TOF MS. Accuracy of the method was determined with 20 positive and 20 negative control experiments using bacteria-spiked whole blood samples. Specificity was evaluated with multi-species spiked whole blood samples.

With high sensitivity, accuracy and specificity, the present method is promising for BSI diagnosis. To demonstrate this concept, the method was tested with clinical *E. coli* or *S. aureus* positive BC bottles provided by a local hospital (Hôpital du Valais, Sion, Switzerland), where the bacteria were successfully identified. Owing to the method’s high sensitivity, the BC time needed for clinical diagnosis can be reduced. As a proof of concept, human whole blood spiked with a low initial concentration (10^2^ or 10^3^ cells per mL) of *E. coli* was cultured in commercial BacT/Alert® FA Plus BC bottles (bioMérieux, Inc., Durham, NC) and analysed using the developed method after different culture times. For both concentrations, *E. coli* was successfully identified after 4 hours of culture.

## Results and discussion

### Direct MALDI-TOF MS fingerprinting of pure bacteria with high sensitivity to build a library of bacteria reference mass spectra

The sensitivity of MALDI-TOF MS for direct bacteria fingerprinting was first investigated using a routine procedure: 1 μL of a bacterial aqueous solution was directly deposited on a MALDI target plate and overlaid with a 2,5-dihydroxycinnamic acid (DHB) matrix for MS analysis. Gram-negative *E. coli* and Gram-positive *B. subtilis* and *S. aureus* were chosen as model bacteria. It should be noted that each test in this work was repeated 3–5 times to guarantee reproducibility and one mass spectrum was chosen as a representative example and displayed. The results showed that at least 10^3^ cells of *E. coli* or *B. subtilis* or *S. aureus* per sample spot were required to generate detectable MS signals (Fig. S1, ESI†). In this test, the average diameter of a sample spot on the target plate was 3 mm, while the size of a bacterial cell is typically 0.5 to 10 μm and the diameter of the laser beam used in the MALDI-TOF MS instrument is about 100 μm. As a result, analysis of a low number of bacteria cells was accomplished using many “blind” laser shots, leading to noise accumulation and limited detection sensitivity.

In order to improve the sensitivity, it is necessary to decrease the diameter of the sample spots, thereby increasing the sample surface density and the chance of “efficient” laser shots. Recently, high-density micro-arrays for ultrasensitive MALDI-TOF MS for the realization of single cell analysis were developed, where each sample spot was only 100 μm in diameter.^20^ In the present work, the sample spots were confined within 800 μm. The sample spot size was optimized with the consideration of MB utilization in later experiments. The presence of MBs reduces MALDI efficiency when their surface density is too high. The confined sample spot size was easily achievable in two ways. The first consisted of depositing samples onto the target plate using a droplet-by-droplet protocol, and the second was based on the utilization of a Bruker MTP AnchorChip target plate (see Experimental section).

**Fig. 1:**
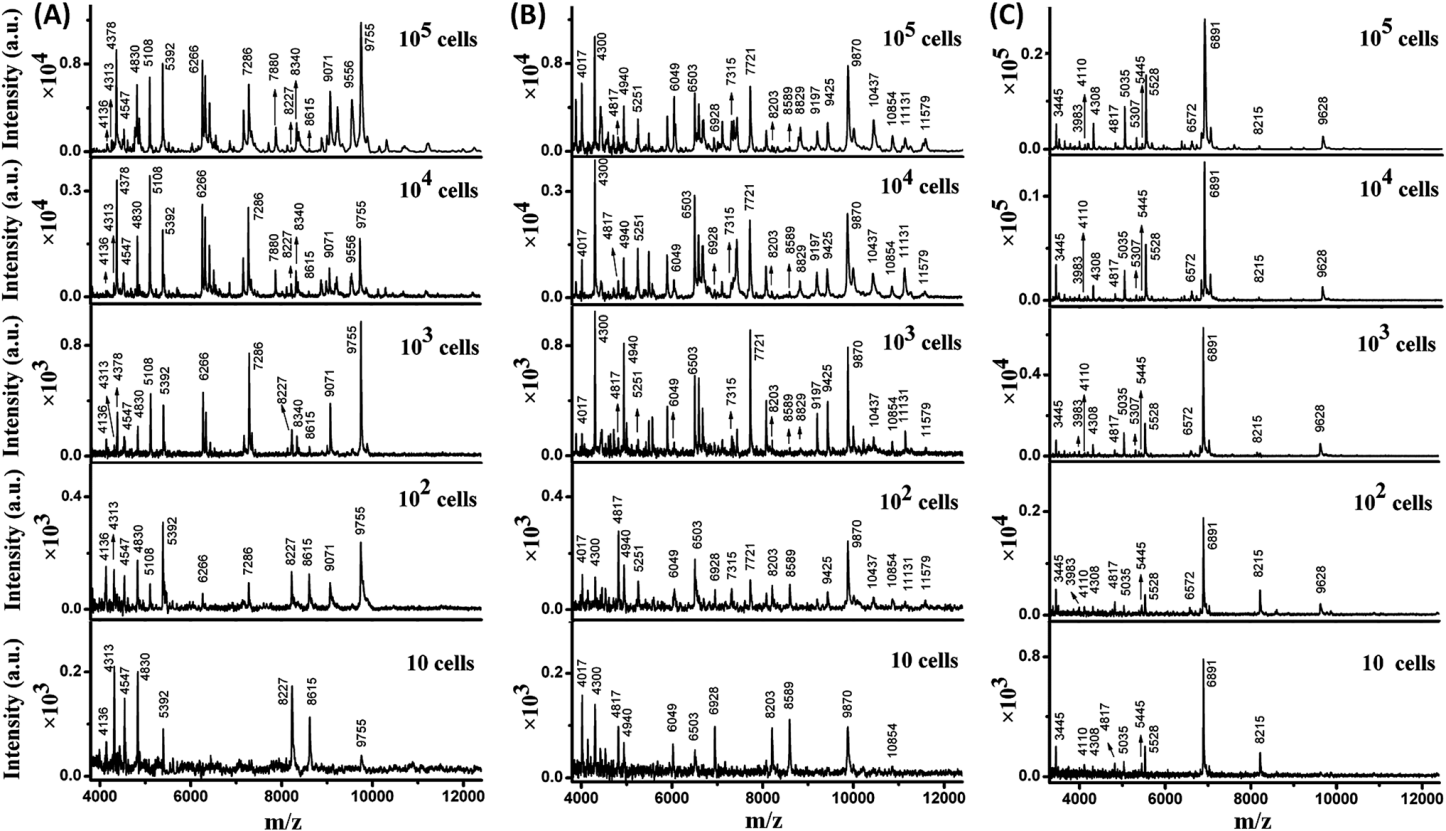
Direct MALDI-TOF MS fingerprinting of intact (A) *E. coli*, (B) *B. subtilis*, and (C) *S. aureus* with reduced sample spot size at different cell numbers: 10^5^ cells (10^8^ cells per mL × 1 μL), 10^4^ cells (10^7^ cells per mL × 1 μL), 10^3^ cells (10^6^ cells per mL × 1 μL), 10^2^ cells (10^5^ cells per mL × 1 μL), and 10 cells (10^4^ cells per mL × 1 μL).

Taking advantage of the small sample spots, the sensitivity of MALDI-TOF MS was significantly enhanced. The MS fingerprint of *E. coli* was successfully obtained from as few as 10 to 10^2^ cells (Fig. 1A). In the case of 10 cells, the cell number was confirmed by counting under a microscope before matrix deposition. Although the peak number decreased with the decrease in *E. coli* cell number, peaks at 4136, 4313, 4547, 4830, 5392, 8227, 8615, and 9755 *m*/*z* (mass-to-charge ratio) appeared reproducibly. Results for *B. subtilis* and *S. aureus* were similar (Fig. 1B and C), with characteristic peaks at 4017, 4300, 4817, 4940, 6049, 6503, 6928, 8203, 8589, 9870, and 10 854 *m*/*z* appearing reproducibly for *B. subtilis*, and peaks at 3445, 4110, 4308, 4817, 5035, 5445, 5528, 6891, and 8215 *m*/*z* appearing reproducibly for *S. aureus*. These peaks corresponded to different intracellular proteins (mainly ribosomal proteins), which could serve as biomarkers to characterize the bacterial species.^24^,^25^

Thus, a library of bacteria reference mass spectra was built with the current data: 3 species (*E. coli*, *B. subtilis*, *S. aureus*) at 5 different cells numbers (10, 10^2^, 10^3^, 10^4^, 10^5^) with 3–5 repetitions in each case. Representative spectra are shown in Fig. 1. It is worth noting that existing commercial databases for bacteria identification are normally built by collecting reference spectra from large cell numbers, which can provide fruitful peak information for highly confident identification.^11^,^26^ However, in such a case, samples with high bacterial abundance are needed to provide spectra with good similarity to the reference for successful identification. Therefore, a large sample volume or long bacteria culture time is required. In this work, reference spectra were collected from a small number of cells as well and this was demonstrated to be sufficient for bacteria identification.

### Identification of bacteria based on mass spectra pattern matching

For pattern matching-based bacteria identification, the experimental spectrum obtained from a blood sample was compared with every library spectrum, and spectral similarity scores were calculated. Bacteria were identified according to the highest score. The similarity score between two mass spectra (i and j) was calculated by the often-used cosine correlation method,^21^ defined as:
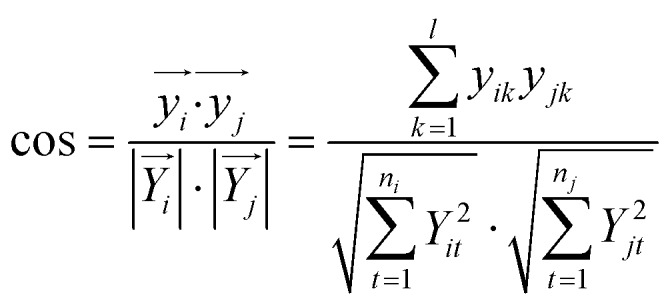
where *y* is the normalized intensity of a peak appearing in both spectrum i and spectrum j (an identical peak), *l* is the number of identical peaks in the two spectra, *Y* is the normalized intensity of a peak appearing in a spectrum and *n* the number of peaks in a spectrum. Only peaks with S/N ≥ 3 were considered. Peaks appearing in different spectra with Δ(*m*/*z*)/(*m*/*z*) ≤ 1000 ppm were considered as identical peaks. A tolerance of 1000 ppm was chosen according to the low resolving power of linear mode TOF analysis.

The scoring method was first adopted to calculate the similarity between the reference mass spectra as illustrated in the previous section (not only the spectra shown in Fig. 1, but also their repetitions). The similarity scores were calculated for three groups: (i) the reference spectra obtained from the different bacteria (867 scores obtained); (ii) the reference spectra obtained from the same bacteria, but at different cell numbers (342 scores obtained); and (iii) the reference spectra obtained from different repetitions of the same bacteria at the same cell number (66 scores obtained). The frequency distribution of the similarity scores in each group is shown in Fig. S2, ESI.† The reference spectra of each bacteria were quite different, with all similarity scores ≤ 0.1 (Fig. S2-A†). When considering the same bacteria at different cell numbers, the similarity scores range from 0 to 1 (Fig. S2-B†). Meanwhile, the reference spectra obtained from different repetitions of the same bacteria at the same cell number were quite similar, with almost all similarity scores ≥ 0.8 (Fig. S2-C†).

Therefore, when the similarity score between a sample spectrum and a reference spectrum is ≥0.8, we can assume that the similarity is very high, as high as the similarity from different duplicated standard samples, and thereby the bacteria in the sample can be identified as the reference one. A score of ≥ 0.8 was considered as the threshold for a successful identification.

### Immunoaffinity MALDI-TOF MS for bacteria identification from spiked human blood serum and whole blood

**Scheme 1:**
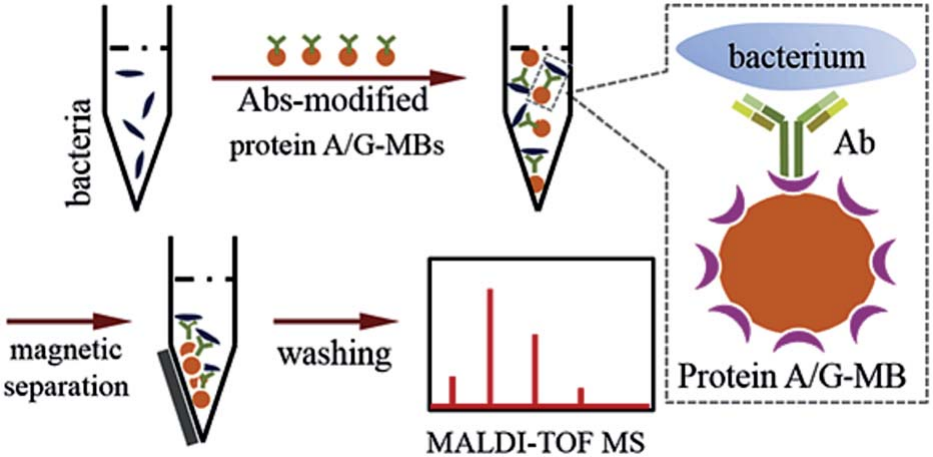
Schematic representation of the immunoaffinity MALDI-TOF MS procedure.

When bacteria are present in a complex medium, *i.e.* blood serum or whole blood, it is hard to efficiently perform direct MALDI-TOF MS identification due to interference from that medium. Thereby, a separation or extraction process is needed before MS detection in order to purify and concentrate the target bacteria. Due to the high specificity of immunoassays and the convenience of magnetic separation, MBs modified with anti-bacterial Abs were chosen for the enrichment and extraction of the target bacteria. This immunoaffinity MALDI-TOF MS approach is shown in Scheme 1.

First, protein A/G-coated MBs were mixed with excess anti-bacterial Abs to form Abs-MBs conjugates and avoid an affinity interaction between MBs and the components of the blood serum or whole blood. The recombinant protein A/G on the surface of MBs contains 6 binding domains (4 from protein A, 2 from protein G) to the Fc regions of the IgG isotype Abs.^27^ A blocking buffer (PBST + 1% BSA) was used to further minimize non-specific adsorption. Prior to the addition of Abs-MBs for bacteria extraction, sample pre-treatment was conducted: blood serum samples were diluted 10 times with PBST buffer to reduce potential interference from serum proteins; whole blood samples were treated with a stepwise centrifugation protocol to purify the bacterial cells (see further details in the Experimental section).^28^ Neither the protein A/G-MBs nor the Abs generate any signal in the mass range (2000–20 000 *m*/*z*) for bacteria identification (Fig. S3, ESI†). Therefore, all of the obtained magnetic mixture, including the MBs, Abs and bacterial cells, was transferred onto a target plate *via* the droplet-by-droplet deposition protocol for MALDI-TOF MS detection, followed by identification based on spectra pattern matching.

To maximize the efficiency of bacteria enrichment, experimental conditions were optimized by comparing the quality of the mass spectra obtained with various amounts of Abs-MBs (Fig. S4, ESI†). An *E. coli*-spiked (10^4^ cells per mL) aqueous sample was chosen as the test sample. When amount of Abs-MBs was too low, a small amount of bacteria was captured and the obtained spectrum was of poor quality. In contrast, when the amount of Abs-MBs too high, this reduced MALDI efficiency. Finally, 50 μg of Abs-MBs was chosen as the optimal condition.

Before analysis of the bacteria-spiked samples, pure blood serum and whole blood without any bacteria were analysed as negative controls using the immunoaffinity MALDI-TOF MS method. No peaks were observed in the mass range of 2000–20 000 *m*/*z* (Fig. S5, ESI†), demonstrating the good specificity of the immunoaffinity extraction. The blood serum and whole blood spiked with a high concentration (10^8^ cells per mL) of *E. coli*, *B. subtilis* or *S. aureus* were then analysed. By pattern matching, it was found that the resulting mass spectra showed the highest similarity to the reference spectra of the corresponding bacterial species at 10^5^ cells. The similarity scores for the blood serum and whole blood samples were 0.953 ± 0.025 and 0.966 ± 0.020 for *E. coli*, 0.976 ± 0.009 and 0.959 ± 0.020 for *B. subtilis* and 0.993 ± 0.005 and 0.986 ± 0.006 for *S. aureus*, as listed in Table S3, no. 1–18, ESI.† A representative spectrum obtained from each sample and a comparison with the corresponding reference spectrum are shown in Fig. S6, ESI.†

**Fig. 2:**
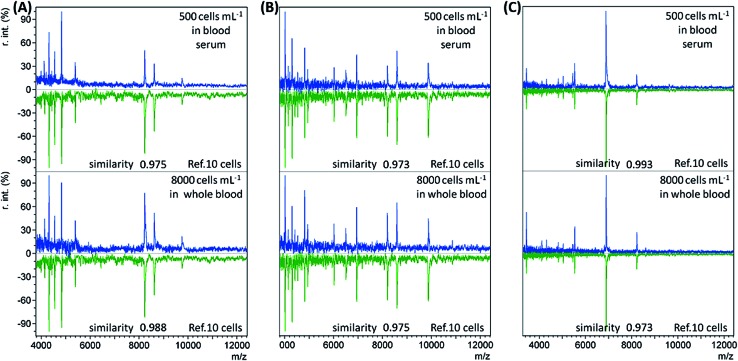
Immunoaffinity MALDI-TOF mass spectra (in blue) for a low concentration of (A) *E. coli*, (B) *B. subtilis*, and (C) *S. aureus* in blood serum (500 cells per mL) or whole blood (8000 cells per mL), and comparisons with the reference spectra (in green) of the corresponding species at 10 cells with similarity scores calculated using the cosine correlation method (r.int: relative intensity).

When the bacterial concentration was gradually decreased, it was found that bacteria with an abundance as low as 500 cells per mL in blood serum (concentration before dilution) and 8000 cells per mL in whole blood could still be identified. The resulting mass spectra displayed the highest similarity to the reference spectra of the corresponding bacterial species at 10 cells, with all similarity scores > 0.9 (Table S3, no. 19–36, ESI†). Representative sample spectra and comparison with the corresponding reference are shown in Fig. 2 (see Table S1, ESI† for a detailed peaks list). For blood serum samples with 500 bacterial cells per mL, the concentration was only 50 cells per mL after dilution. Considering that only 1 mL of sample was used, it is reasonable that the obtained sample spectra should match well with the reference spectra for 10 cells. The LODs for the whole blood samples were not as good as those for the blood serum samples. The main reason for this is the loss of bacterial cells during the sample pre-treatment process.

The accuracy of the immunoaffinity MALDI-TOF MS method was then evaluated. 20 whole blood samples spiked with random concentrations of *S. aureus* (>8000 cells per mL) were analysed using anti-*S. aureus* Abs-MBs. *S. aureus* was successfully identified from 19 samples, as the resulting spectra displayed the highest similarity (score > 0.8) to the reference spectra of *S. aureus* (Table S3, no. 37–56, ESI†). For the remaining sample, the resulting spectrum also best matched with the reference spectrum of *S. aureus*, but the similarity score was only 0.714. Another 20 whole blood samples spiked with random concentrations (>8000 cells per mL) of *E. coli* or *B. subtilis* were analysed as negative controls, also using anti-*S. aureus* Abs-MBs. No peaks were observed in the resulting spectra. Therefore, the present method enables accurate identification of bacteria from blood samples.

In many cases, patients are infected with polymicrobial BSI (*i.e.* BSI caused by more than one pathogen).^29^ Normal MALDI-TOF MS is not efficient for samples containing several bacterial species. Proteins or membrane lipids from different species can make the resulting mass spectra too complicated to be analysed. A subculture, taking hours to days, is often conducted to provide pure bacterial cultures for identification. The immunoaffinity MALDI-TOF MS method is based on a highly specific interaction between the bacteria and anti-bacterial Abs. Therefore, it would be possible to identify target bacteria from multi-species infected samples without a subculture. To prove this concept, three groups of whole blood samples spiked simultaneously with *S. aureus*, *E. coli*, and *B. subtilis* were analysed with anti-*S. aureus* Abs-MBs. The compositions of the samples were: (A) 10^5^ cells per mL *S. aureus*, 10^4^ cells per mL *E. coli*, 10^4^ cells per mL *B. subtilis*; (B) 10^5^ cells per mL *S. aureus*, 10^5^ cells per mL *E. coli*, 10^5^ cells per mL *B. subtilis*; and (C) 10^5^ cells per mL *S. aureus*, 10^7^ cells per mL *E. coli*, 10^7^ cells per mL *B. subtilis*. As shown in Table 1 (and Table S3, no. 57–65, ESI†), *S. aureus* was correctly identified from all samples. When the concentrations of both *E. coli* and *B. subtilis* were more than 500 times the *S. aureus* concentration, *S. aureus* could no longer be identified (data not shown). The extra high concentrations of *E. coli* and *B. subtilis* decreased the chance of *S. aureus* binding with MBs. Overall, the immunoaffinity MALDI-TOF MS method allows bacteria identification from multi-species infected blood samples when the relative concentrations of interference bacteria are not too high. This method shows potential for polymicrobial BSI diagnosis.

**Table 1:** A list of spectra pattern matching results for three groups of multi-species spiked whole blood samples^*a*^

Sample	Reference spectra	Similarity score
A-1	10 *S. aureus* cells	0.990
A-2	10^2^*S. aureus* cells	0.993
A-3	10^2^*S. aureus* cells	0.991
B-1	10 *S. aureus* cells	0.991
B-2	10 *S. aureus* cells	0.985
B-3	10 *S. aureus* cells	0.992
C-1	10^2^*S. aureus* cells	0.969
C-2	10 *S. aureus* cells	0.982
C-3	10 *S. aureus* cells	0.970

^*a*^1–3: three independent repetitions.

To sum up, a sensitive method has been demonstrated for the accurate and specific identification of bacteria from spiked blood samples by combining a highly efficient immunoaffinity enrichment/separation and optimized MALDI-TOF MS detection. The method requires only 1 mL of sample. The LOD for whole blood (8000 cells per mL) is still higher than the bacterial concentration found in adult BSI patients and direct bacteria identification from patient blood remains difficult. However, the much shorter BC period can be expected to magnify the bacterial concentration to a detectable level. Moreover, this method is particularly suitable for clinical diagnosis in child patients, as BSI in child patients normally has a much higher microorganism concentration (often >100 CFU mL^–1^).^3^,^17^

### Bacteria identification from BCs

In standard clinical diagnosis, blood samples collected from patients are cultured in BC bottles to investigate the presence or absence of bacteria. If bacteria are present, they can proliferate in the bottles during the culture process. When the abundance of bacteria is high enough, the bottles turn positive automatically. For example, positive BacT/Alert® bottles indicate a high bacterial abundance by a means of a colour change on the bottom. Before the final definitive identification step (*e.g.* biochemical phenotyping), isolates from the positive bottles undergo gram staining, plating, and subculture to further magnify bacterial concentrations or to separate different bacteria.^3^

**Fig. 3:**
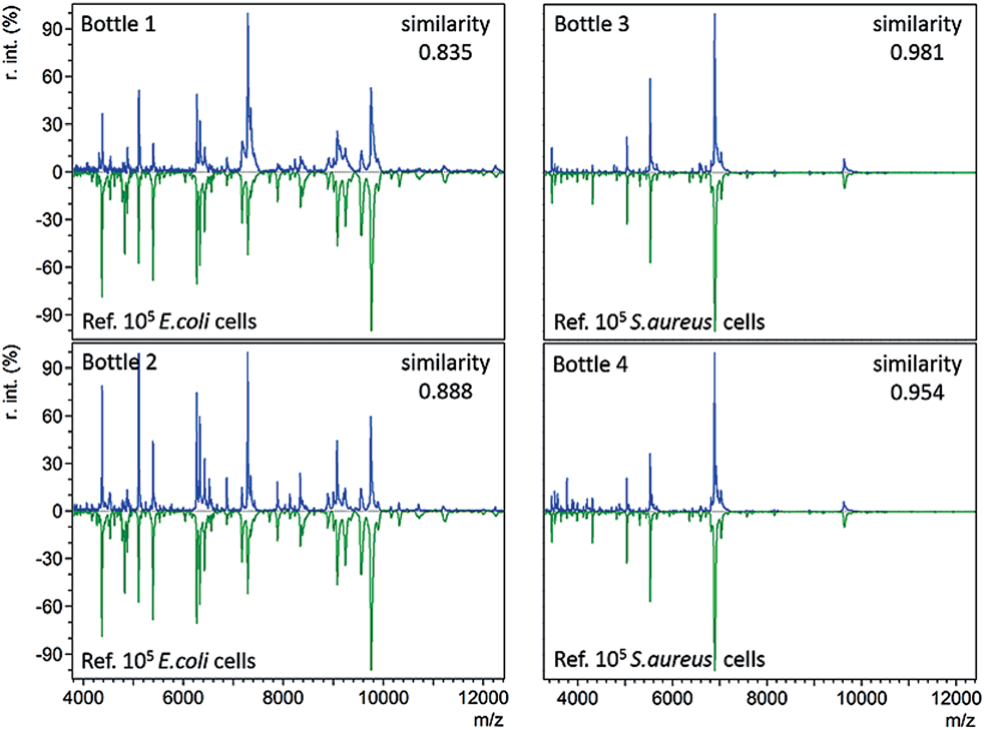
Immunoaffinity MALDI-TOF mass spectra (in blue) obtained from four positive BC bottles and comparison with the reference spectra (in green) with similarity scores calculated using the cosine correlation method.

The developed immunoaffinity MALDI-TOF MS method was used for bacteria identification directly from four clinical positive BC bottles. Two of the bottles (bottle 1 and 2) were *E. coli* positive. Another two (bottle 3 and 4) were *S. aureus* positive. 1 mL of positive culture liquid was taken from each bottle and analyzed with anti-*E. coli* or anti-*S. aureus* Abs-MBs. Consequently, *E. coli* was correctly identified from bottle 1 and 2, as the resulting mass spectra displayed the highest similarity to the reference spectra of 10^5^*E. coli* cells, with similarity scores of 0.835 and 0.888, respectively (Table S3, no. 66–67, ESI†). *S. aureus* was successfully identified from bottle 3 and 4, scoring 0.981 and 0.954, respectively (Table S3, no. 68–69, ESI†). The resulting mass spectra and a comparison with the reference spectra are shown in Fig. 3 (see Table S2, ESI† for a detailed peaks list). These results indicate that the present method can identify bacteria directly from positive BCs, without the need for a subculture. Therefore, this method can shorten the time for BSI diagnosis.

For all four positive BC bottles, the resulting mass spectra were comparable to the reference spectra with large numbers of *E. coli* or *S. aureus* cells (10^5^ cells), as shown in Fig. 3. This implies that the bacterial concentrations are relatively high when the BC bottles turn positive. As the LOD of the present method in whole blood samples is 8000 cells per mL, it should allow bacteria identification before the bottles turn positive. In such a case, the BC time required for diagnosis could be reduced. To demonstrate this concept, a time-step test was conducted to investigate the influence of BC time on the identification result.

5 mL of whole blood collected from a healthy adult was spiked with *E. coli* at an initial concentration of either 10^2^ or 10^3^ cells per mL and cultured in a BacT/Alert® FA Plus BC bottle. The initial concentrations of 10^2^ and 10^3^ cells per mL were chosen in accordance with the normal bacterial concentration found in BSI patients. For each concentration, four BC bottles (I, II, III, IV) were prepared in parallel. For two of these bottle (I, II), 1 mL of culture liquid was removed and analyzed using the immunoaffinity MALDI-TOF MS method every 2 hours. The remaining two bottles (III, IV) were left untouched in order to observe when they would turn positive automatically (indicated by a colour change on the bottom).

**Fig. 4:**
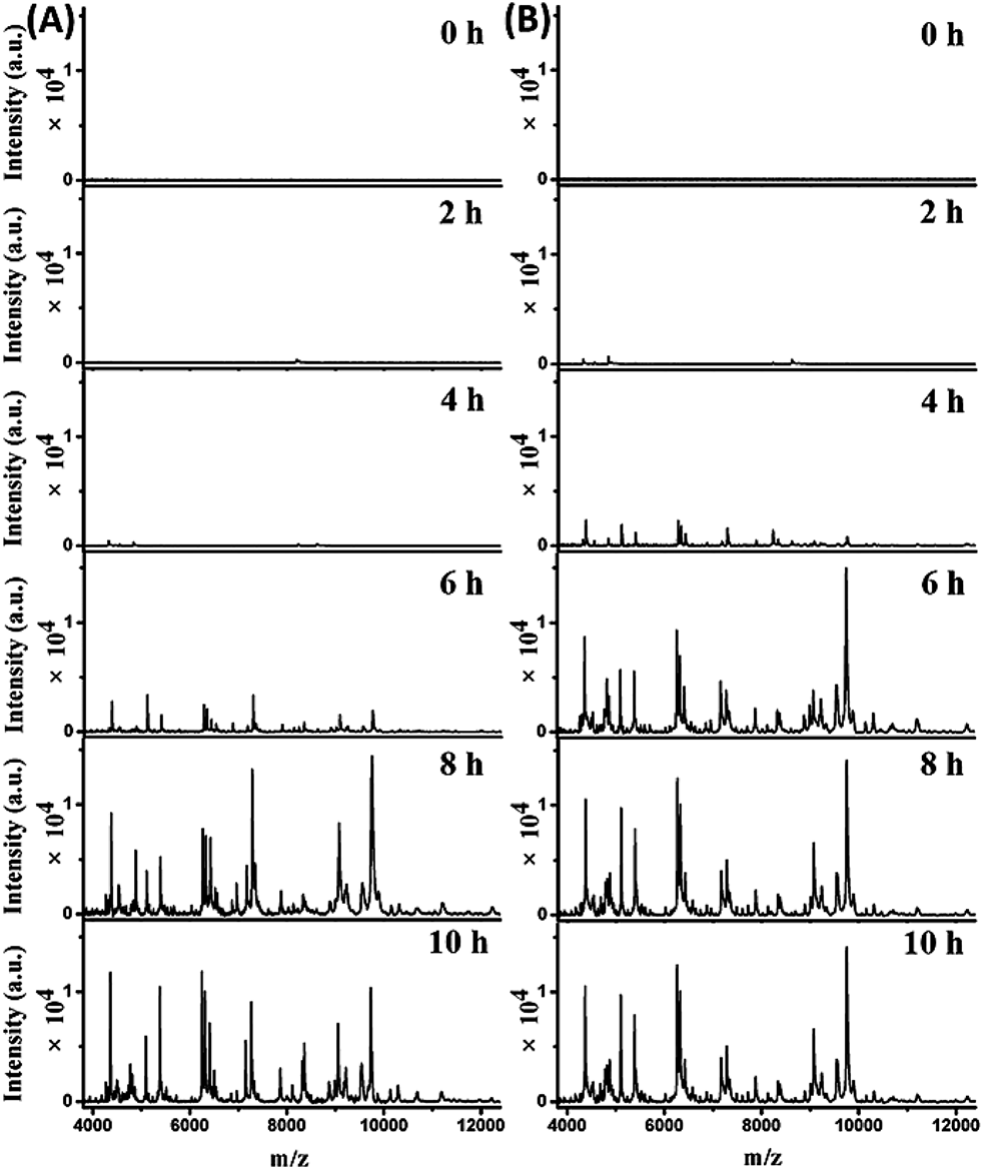
Immunoaffinity MALDI-TOF mass spectra obtained from BC bottles with initial *E. coli* concentrations of (A) 10^2^ cells per mL and (B) 10^3^ cells per mL in 5 mL of blood after different BC times: 0 h, 2 h, 4 h, 6 h, 8 h and 10 h.

The mass spectra obtained from the two parallel bottles loaded with whole blood containing 10^2^ cells per mL *E. coli* are shown in Fig. 4A (bottle I) and S7-A, ESI† (bottle II). The best pattern matching results for these spectra are displayed in Fig. S8 and Table S3, no. 70–75, ESI.† At the beginning (0 hour of BC), no characteristic peaks were detected, as the *E. coli* concentration was below the LOD. After 2 hours, one peak at *m*/*z* 8226 appeared for both bottles. Considering a tolerance of 1000 ppm, this should be identical to the peak of *m*/*z* 8227 in the reference spectrum for 10 *E. coli* cells (Fig. S8, A-I, A-II, ESI†). After 4 hours, more peaks were detectable, with a similarities of 0.927 (bottle I) and 0.867 (bottle II) to the reference spectrum for 10 *E. coli* cells (Fig. S8, B-I, B-II, ESI†). Good quality mass spectra were obtained after 6 hours, showing similarities of 0.959 and 0.876 to the reference spectrum for 10^4^ and 10^3^*E. coli* cells, respectively (Fig. S8, C-I, C-II, ESI†). After 8 hours, the obtained spectra were similar to the reference spectrum for 10^5^*E. coli* cells, with similarities of 0.913 and 0.897 for bottle I and II, respectively (Fig. S8, D-I, D-II, ESI†). The obtained spectra didn't change much when increasing the BC time (*e.g.* 10 h). Therefore, the bacterial abundance was high enough for identification using the developed method after 4 hours of BC. Meanwhile, it was observed that the two untouched bottles turned positive after 10.5–11 hours of BC.

Mass spectra obtained from the two parallel loaded bottles with whole blood containing 10^3^*E. coli* cells per mL are shown in Fig. 4B (bottle I) and S7-B, ESI† (bottle II). The best pattern matching results for these bottles are shown in Fig. S9 and Table S3, no. 76–81, ESI.† The results indicated that *E. coli* could be successfully identified after 2 hours of BC. In comparison, the two untouched bottles turned positive after 9–9.5 hours of BC.

These results demonstrate that the present method is able to identify bacteria before the BC bottles turn positive. Considering the time required for immunoaffinity MALDI-TOF MS analysis (<2 hours), the entire identification process, from blood collection to identification reports, could be completed within 4 to 6 hours, demonstrating this method’s potential application for fast BSI diagnosis.

Utilization of appropriate Abs-coated MBs is critical for the success of the present method. In clinical practice, it is necessary to store Abs against common BSI pathogens, such as *Staphylococcus aureus*, *Staphylococcus epidermidis*, *Staphylococcus hominis*, *Escherichia coli*, *Enterococcus faecium*, *Streptococcus pneumoniae*, *Klebsiella pneumoniae*, *Acinetobacter baumannii*, and *Enterobacter cloacae*.^29^ To guide the selection of suitable Abs, the pathogen species can be empirically pre-judged according to the infection symptoms of a patient, and then tested using the immunoaffinity MALDI-MS method. In extreme cases, identification could always be carried out by scanning the sample with the Abs-MBs bank. To further increase the efficiency, Abs against particular strains of the same bacterial species could also be stored and adopted. Moreover, a library of reference spectra of a greater number of bacterial species, especially common BSI pathogens at different cell numbers, should be built, which could be a future continuation of the current work.

In addition to BSI diagnosis, this method can also be used for the quality control of blood products (whole blood, platelet and erythrocyte concentrates if not pathogen reduced) during storage. For instance, bacterial strains involved in adverse transfusion reactions such as *Staphylococcus epidermis*, *Serratia marcescens* or *Serratia liquefaciens* could be tested in non-pathogen reduced blood products.^30^,^31^

## Conclusions

In this work, an immunoaffinity MALDI-TOF MS method has been developed for the identification of bacteria in human blood samples at the species level. By comparing the resulting sample spectra with references in a self-built library, bacteria can be correctly identified from whole blood samples with LOD of 8000 cells per mL (1 mL). The method is able to identify bacteria from polybacterial blood samples, showing potential in polymicrobial BSI diagnosis. It has also been successful in direct bacteria identification from clinical positive BC bottles. A time-step test suggests that bacteria can actually be identified from BC bottles even before they turn positive. The culture time required for diagnosis is also significantly reduced. With this method, the entire BSI diagnosis can be finished within half a day.

## Experimental

### Materials

Goat polyclonal IgG isotype Abs against *E. coli*, rabbit polyclonal IgG isotype Abs against *B. subtilis* and rabbit polyclonal IgG isotype Abs against *S. aureus* were purchased from Abcam plc (Cambridge, UK). Pierce™ protein A/G-coated MBs were purchased from Thermo Fisher Scientific Inc. (Waltham, Massachusetts, USA). Human blood serum was purchased from Bioreclamation LLC (New York, USA). Human whole blood was donated by a healthy female in her 20s and was collected by the Transfusion Interrégional CRS, Lausanne, Switzerland. Positive BC bottles with clinical BSI patients' blood and un-used BacT/Alert® FA Plus BC bottles were provided by Hôpital du Valais (Sion, Switzerland). Acetonitrile (HPLC grade) was purchased from Aventor Performance Materials (Center Valley, PA, USA). Trifluoroacetic acid (TFA) (99.0%) was obtained from Acros Organics (New Jersey, USA). 2,5-Dihydroxycinnamic acid (DHB), Tween-20, bovine serum albumin (BSA), disodium hydrogen phosphate dodecahydrate (≥99.0%), sodium phosphate monobasic dihydrate (≥99.0%), and sodium chloride (NaCl) (≥99.5%) were all purchased from Sigma-Aldrich (St. Gallen, Switzerland). Deionized (DI) water (18.2 MΩ cm) was purified by an alpha Q Millipore system (Zug, Switzerland), and used in all aqueous solutions.

### Bacterial cell culture

*E. coli* strain DH5α (obtained from Life Technologies) was grown as a pre-culture in 2 mL of Luria-Bertani (LB) medium (Sigma-Aldrich) at 37 °C for 6 h with continuous shaking at 250 rpm. 100 μL of the *E. coli* DH5α pre-culture was added into 3 mL LB and incubated overnight at 37 °C with continuous shaking.

*B. subtilis* strain ATCC6633 (obtained from the American Type Culture Collection, Rockville, Md.) and *S. aureus* strain Col (provided by Lausanne University) were grown in 20 mL of LB medium in 100 mL Erlenmeyer flasks. Incubation was carried out at 37 °C for 16 h with continuous shaking at 180 rpm.

The concentration of bacteria in the culture media was determined by measuring the optical density at 600 nm by UV-visible absorption spectroscopy.

### Direct MALDI-TOF MS fingerprinting of intact bacteria

Bacterial cells were separated from the growing media by centrifugation (13 000 rpm × 3 min), and washed three times with DI water. Finally, the resulting cellular pellet was resuspended in DI water at a concentration of 10^8^ cells per mL. Bacteria solutions with different concentrations were obtained by dilution with DI water. 1 μL of each solution was deposited on a MALDI target plate, and dried at room temperature (RT). For sample deposition, three different protocols were conducted. In the first (the routine procedure), the whole 1 μL of solution was deposited on a Bruker ground steel target plate, and the dried sample spot size was about 3 mm in diameter. In the second, 1 μL of solution was deposited on the target plate with four repetitions (0.25 μL in each repetition), droplet-by-droplet, in order to keep the sample spot as small as possible (<0.8 mm in diameter). In the third, a Bruker MTP AnchorChip target plate was employed, where the dried sample spot from 1 μL of a bacteria solution could be confined within a well that was 0.8 mm in diameter. DHB matrix (1 μL, 10 mg mL^–1^ in *V*_acetonitrile_/*V*_water_/*V*_TFA_ 50/49.5/0.1) was added to cover the dried sample spots with the corresponding protocol for MALDI-TOF MS analysis. Each test was repeated 3–5 times. A library of bacteria reference mass spectra was built from the spectra obtained with the reduced sample spot size.

### Bacteria identification from human blood samples

50 μg of protein A/G (∼50.5 kDa)-coated MBs (1 μm in diameter) were washed twice with PBST buffer and dispersed in 50 μL of PBST buffer. An excess of Abs (2 μL × 2 mg mL^–1^) was added to bind with MBs. The mixture was incubated for 30 min at RT with continuous shaking. The obtained Abs-MBs were collected using a magnetic stand and blocked with PBST buffer containing 1% BSA. BSA was used to minimize non-specific adsorption during the following immunoassay.

Blood serum samples or whole blood samples spiked with different concentrations of bacteria were prepared. To reduce interference from the blood serum or whole blood, sample pre-treatment was conducted. For the spiked serum samples, 0.1 mL of each sample was diluted with PBST buffer to 1 mL before the addition of Abs-MBs. For the spiked whole blood samples, 1 mL of each sample was diluted 6 times with DI water and then centrifuged at 140*g* for 10 min to sediment the blood cells. The supernatant was recovered and mixed with 2 mL of DI water to lyse any residual erythrocytes. After centrifugation at 2000*g* for 5 min, a bacteria pellet was obtained, which was washed with 1 mL DI water and finally resuspended in 300 μL of PBST buffer. For the tests using the clinical positive BC bottles, 1 mL of culture fluid was taken from each bottle with the help of a sterile syringe and the bacteria were purified using the same stepwise centrifugation protocol presented above.

Afterwards, 50 μg of Abs-MBs were added to capture the bacterial cells. After incubation at 37 °C for 30 min with continuous shaking, the MBs were collected and washed twice with a PBST buffer containing 0.1% BSA (1 mL) and once with DI water (1 mL). The samples were then completely deposited onto a normal MALDI target plate *via* a droplet-by-droplet protocol or deposited onto an AnchorChip target plate. After drying at RT, the sample spot was overlaid with DHB matrix for MALDI-TOF MS detection.

### Time-step test during the BC process

5 mL of human whole blood was spiked with *E. coli* cells with an initial concentration of either 10^2^ or 10^3^ cells per mL. The spiked blood was injected into a BacT/Alert® FA Plus BC bottle and cultured at 37 °C with continuous shaking. For each concentration, four BC bottles were prepared in parallel. For two of these bottles, during the culture process, 1 mL of culture liquid was removed and analyzed with the proposed immunoaffinity MALDI-TOF MS method. The test was conducted every 2 hours (0 h, 2 h, 4 h, 6 h, 8 h and 10 h). The remaining two bottles were left untouched and observed until they turned positive (indicated by an automatic colour change on the bottom of the bottle, from grey-green to bright yellow).

### MS detection and data analysis

MALDI-TOF MS analysis was performed on a Bruker MicroFlex LRF in linear positive mode. The instrumental parameters were: 65% laser intensity, accumulation from 500 laser shots, 10.3× detector gain and 400 ns delayed extraction time. Mass spectra peak picking was performed with the mMass Open Source Mass Spectrometry Tool (http://www.mmass.org). The similarity scores obtained during the pattern matching process were calculated using the cosine correlation method. All of the mathematical calculations were conducted with “R” from the R Foundation for Statistical Computing (; http://www.R-project.org). Only peaks with S/N ≥ 3 were considered, with a mass tolerance of 1000 ppm. The similarity scores of each sample spectrum compared to each library spectrum were calculated, and the identification results were determined by the highest score. A score ≥ 0.8 was the threshold for successful identification.

### Safety consideration

All practical activities with pathogenic bacterial strains were conducted in a biosafety level 2 (P2) laboratory. Laboratory coats and gloves were worn during the entire activity process and they were never worn outside the laboratory. All bacterial waste was disposed properly according to the safety guidelines. When activities were finished, instruments, facilities and benches were wiped down with 70% ethanol. Hands were washed with soap and water before leaving the laboratory.

### Live subject statement

All of the blood samples were collected under signed consent of the donors. No research on genetic material was carried out. Therefore, these samples were in agreement with the “Loi fédérale relative à la recherche sur l'être humain, LRH – RS 810.30” and the “Ordonnance relative à la recherche sur l'être humain, ORH – RS 810.301”.

## Supplementary Material

Supplementary information
