# Supplementary material for: Sensitive and fast identification of bacteria in blood samples by immunoaffinity mass spectrometry for quick BSI diagnosis
Source: Chem Sci. 2016 Jan 26;7(5):2987–95. doi: 10.1039/c5sc04919a (PMC6004777; doi:10.1039/c5sc04919a)
Supplement: Supplementary file 1 [file SC-007-C5SC04919A-s001.pdf]

Electronic Supplementary Material (ESI) for Chemical Science.
This journal is © The Royal Society of Chemistry 2016

Electronic Supplementary Information (ESI) for:

Sensitive and fast identification of bacteria in blood samples by immunoaffinity mass spectrometry for quick BSI diagnosis

Yingdi Zhu[a], Liang Qiao[a,e], Michel Prudent[b], Alexandra Bondarenko[a], Natalia Gasilova[a], Siham Beggah Möller [c], Niels Lion[b], Horst Pick[d], Tianqi Gong[e], Zhuoxin Chen[e], Pengyuan Yang[e], Lysiane Tissières Lovey,[f] and Hubert H. Girault[a*]

a. Laboratoire d'Electrochimie Physique et Analytique, École Polytechnique Fedérale de Lausanne, Rue de l'industrie 17, CH-1951 Sion, Switzerland
b. Transfusion Interrégionale CRS, Laboratoire de Recherche sur les Produits Sanguins, CH-1015 Lausanne, Switzerland
c. Department of Fundamental Microbiology, University of Lausanne, CH-1015 Lausanne, Switzerland
d. Laboratoire de Chimie Physique des Polymères et Membranes, École Polytechnique Fédérale de Lausanne, CH-1015 Lausanne, Switzerland
e. Institutes of Biomedical Sciences, Fudan University, Dong'an Road 131, 200032, Shanghai, China
f. Hôpital du Valais, Sion, Avenue du Grand Champsec 80, CH-1951 Sion, Switzerland

*: Corresponding Author: Prof. H. H. Girault, E-mail: hubert.girault@epfl.ch

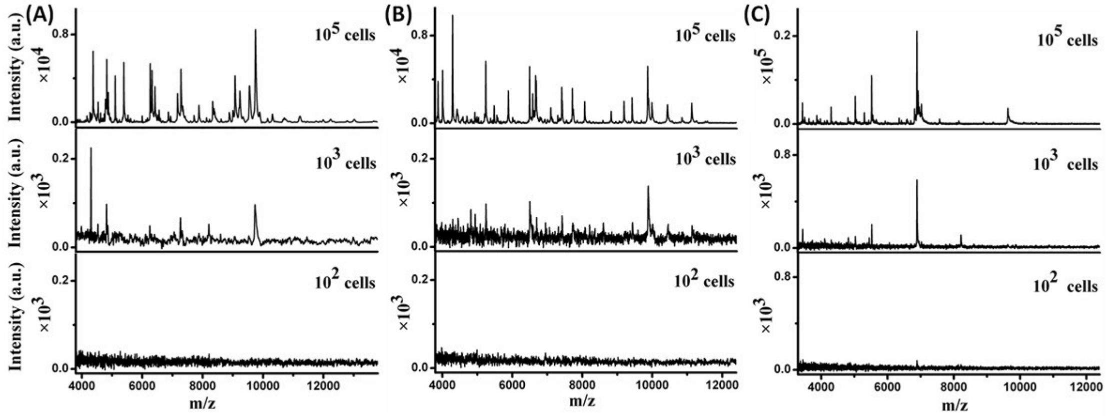

Fig. S1 Direct MALDI-TOF MS fingerprinting of (A) *E.coli*, (B) *B.subtilis*, and (C) *S.aureus* by routine MALDI sample deposition procedure at different cell numbers: 10^5 cells (10^8 cells mL^{-1} × 1 μL), 10^3 cells (10^6 cells mL^{-1} × 1 μL), 10^2 cells (10^5 cells mL^{-1} × 1 μL).

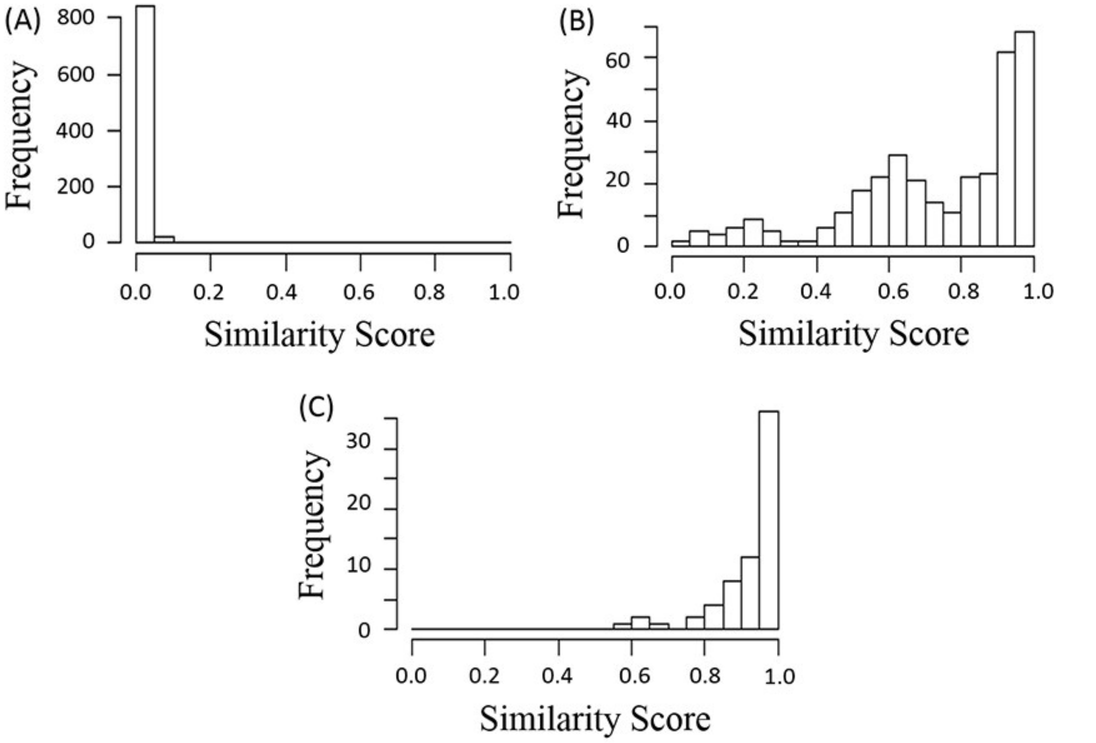

Fig.S2 The frequency distribution of similarity scores between bacteria reference mass spectra for three groups: (A) reference spectra obtained from different bacteria; (B) reference spectra obtained from the same bacteria but at different cell numbers; (C) reference spectra obtained from different repetitions of the same bacteria at the same cell number.

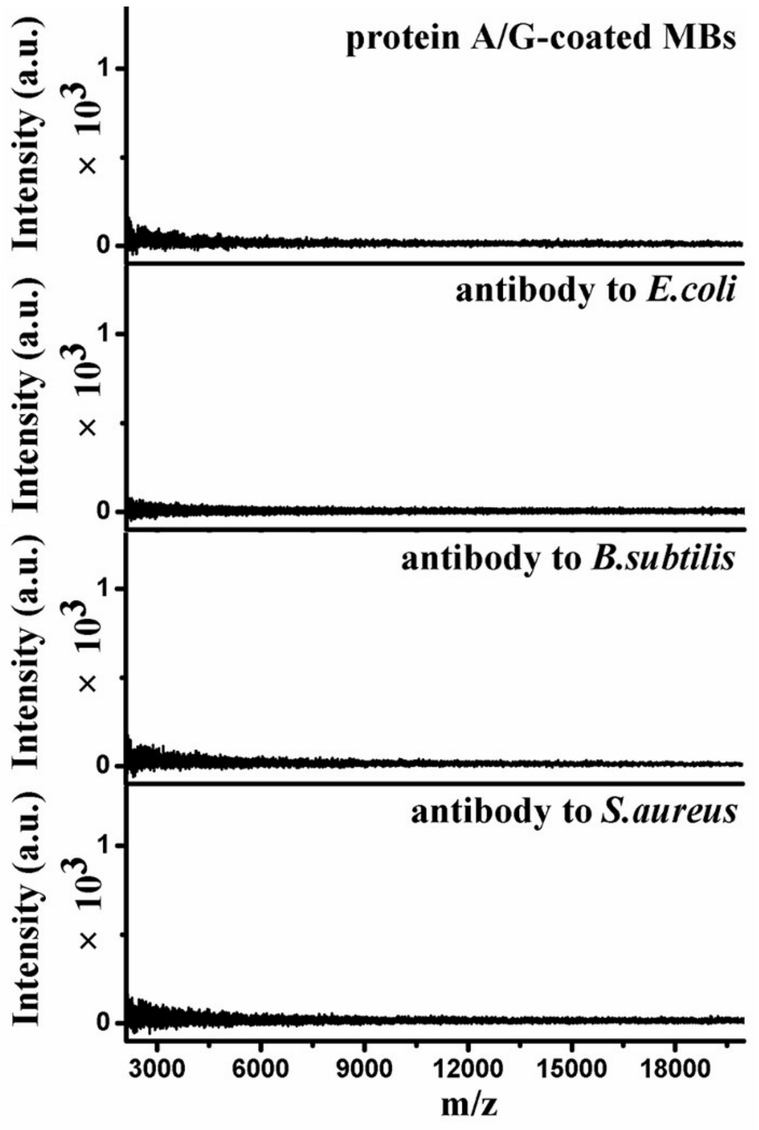

Fig. S3 MALDI-TOF mass spectra of protein A/G-coated magnetic beads (MBs) and anti-bacteria antibodies in the mass range of 2,000-20,000 *m/z*.

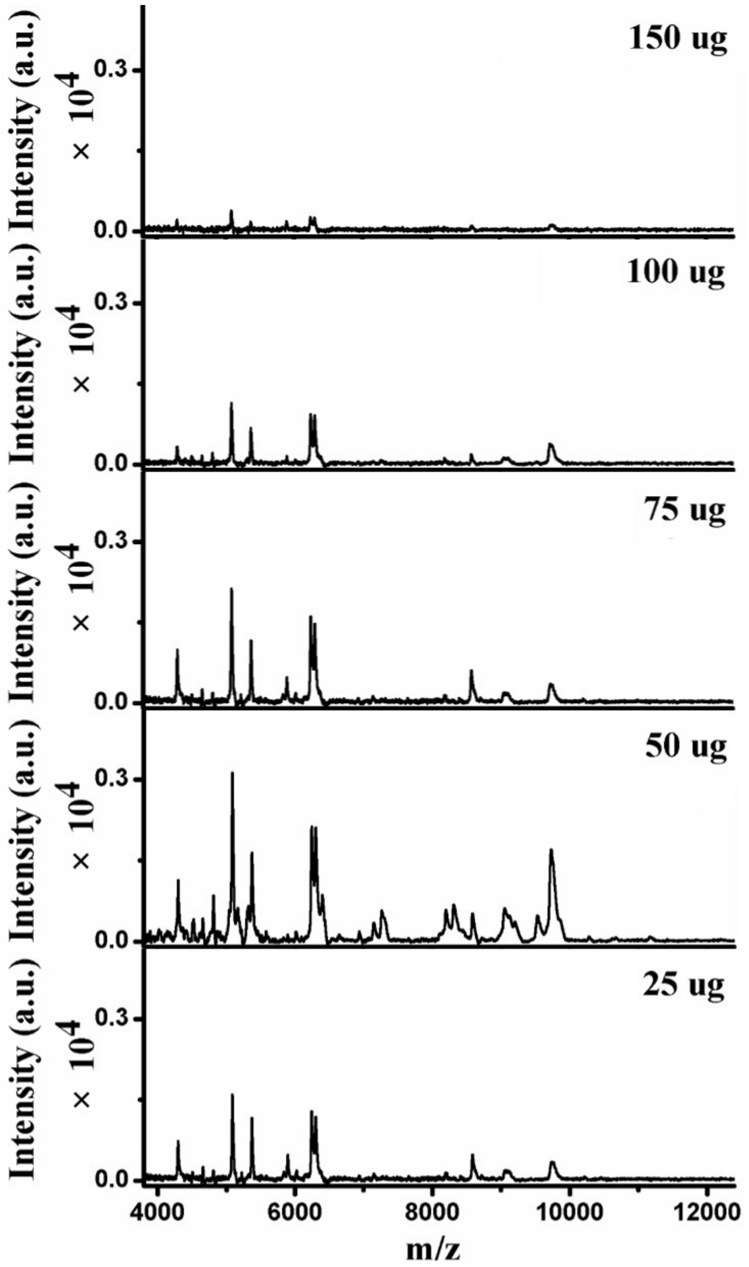

Fig. S4 Immunoaffinity MALDI-TOF mass spectra obtained from aqueous solutions (1 mL) with 10^4 cells mL^{-1} *E.coli* when different amounts of Abs-MBs were used.

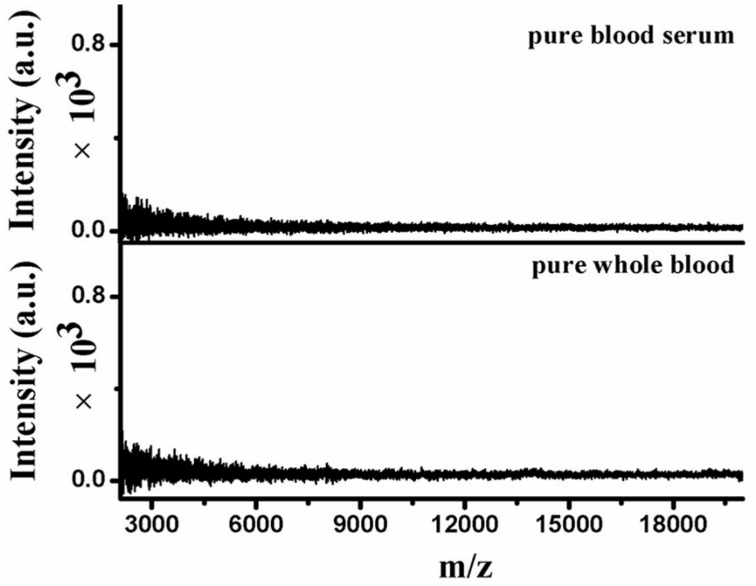

Fig. S5 Immunoaffinity MALDI-TOF mass spectra of pure blood serum and pure whole blood after sample pre-treatment by using anti-*E.coli* Abs-MBs in the mass range of 2,000-20,000 *m/z*.

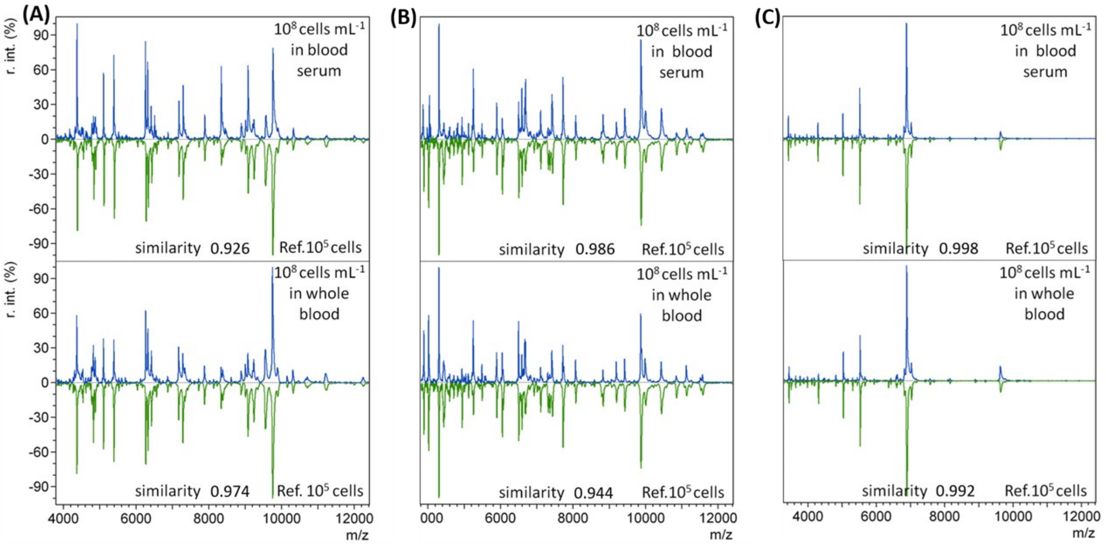

Fig.S6 Immunoaffinity MALDI-TOF mass spectra (in blue) obtained from blood serum and whole blood spiked with 10^8 cells mL^{-1} of (A) *E.coli*, (B) *B.subtilis*, and (C) *S.aureus* and comparison with reference spectra (in green) of corresponding species at 10^5 cells with similarity score calculated by the cosine correlation method. r.int: relative intensity.

Table S1. List of peaks (S/N ≥ 3), including nominal *m/z* values and normalized intensities (I_N), for mass spectra in Fig.2. The matching tolerance is set as 1000 ppm.

(A) for *E.coli* in Fig. 2A

500 cells mL^{-1} in blood serum		8000 cells mL^{-1} in whole blood		reference spectrum at 10 cells	
m/z	I_N	*m/z*	I_N	*m/z*	I_N
4135	29.11	4135	38.68	4136	23.57
4315	71.96	4314	100.00	4313	100.00
4545	44.07	4546	42.19	4547	64.63
4832	100.00	4830	89.84	4830	94.17
5390	30.83	5391	39.76	5392	41.10
8230	51.37	8229	70.66	8227	81.44
8618	32.13	8616	50.65	8615	52.06
9757	11.02	9754	18.57	9755	15.42

(B) for *B.subtilis* in Fig. 2B

500 cells mL^{-1} in blood serum		8000 cells mL^{-1} in whole blood		reference spectrum at 10 cells	
m/z	I_N	*m/z*	I_N	*m/z*	I_N
4019	100.00	4018	100.00	4017	100.00
4145	29.32	4141	23.14	4143	45.50
4302	60.08	4301	62.36	4300	90.47
4418	15.37	4419	21.49	4420	32.15
4531	21.65	4531	20.10	4533	33.06
4817	52.23	4816	84.51	4817	62.14
4940	24.92	4940	35.16	4940	41.84
6048	26.48	6048	37.90	6049	39.70
6504	22.22	6501	31.17	6503	31.16
6929	42.70	6930	60.82	6928	62.55
8202	27.87	8202	52.79	8203	60.21
8590	48.77	8591	66.78	8589	69.87
9870	32.52	9874	44.73	9870	61.63
10856	10.79	10857	10.95	10854	13.81

(C) for *S.aureus* in Fig. 2C

500 cells mL^{-1} in blood serum		8000 cells mL^{-1} in whole blood		reference spectrum at 10 cells	
m/z	I_N	*m/z*	I_N	*m/z*	I_N
3444	23.62	3446	51.92	3445	25.53
4110	8.39	4111	14.25	4110	9.26
4308	10.25	4309	16.90	4308	8.35
4817	12.53	4819	13.36	4817	9.12
5036	15.75	5037	12.02	5035	11.60
5444	19.85	5445	10.82	5445	10.37
5527	32.61	5529	33.85	5528	25.53
6890	100.00	6892	100.00	6891	100.00
8214	17.92	8216	25.83	8215	19.38

Table S2. List of peaks (S/N ≥ 3), including nominal m/z values and normalized intensities (I_N), for mass spectra in Fig.3. The matching tolerance is 1000 ppm.

(A) for mass spectra obtained from Bottle 1,2, and the reference spectrum

mass spectrum from Bottle 1		mass spectrum from Bottle 2		reference spectrum of 10^5 *E.coli* cells	
m/z	I_N	*m/z*	I_N	*m/z*	I_N
4312	12.03	4164	4.30	4162	6.25
4379	37.05	4315	4.61	4268	9.25
4545	10.08	4377	78.84	4313	6.19
4782	5.57	4546	9.85	4378	80.94
4830	5.49	4782	6.99	4547	17.85
4878	15.46	4828	6.42	4618	9.26
5112	52.06	4877	13.64	4780	19.42
5394	18.12	5109	99.31	4830	53.23
5528	3.09	5394	43.72	4876	26.78
6268	49.49	5528	3.89	5108	58.94
6328	32.16	6268	74.65	5392	69.97
6424	14.97	6328	59.80	5526	7.38
6559	3.91	6424	33.17	6036	6.57
6870	9.41	6557	20.48	6266	71.93
7173	17.97	6869	21.10	6326	59.60
7287	100.00	7170	14.96	6422	38.30
7883	4.95	7286	100.00	6559	12.94
8129	4.07	7882	18.47	6869	8.46
8341	9.79	8130	10.87	7170	32.57
8893	9.14	8337	23.64	7286	52.38
9010	9.00	8889	10.92	7880	19.09
9073	24.04	9010	11.78	8132	4.86
9240	14.82	9073	44.35	8340	22.40
9558	13.62	9239	14.37	8891	9.50
9756	51.20	9554	12.67	9013	12.21
9894	7.78	9752	59.24	9071	45.94
10156	2.29	9894	5.33	9242	34.65
10318	2.31	10154	2.40	9556	40.95
10705	2.63	10318	5.41	9755	100.00
11233	4.24	10704	3.92	9896	12.06
12245	4.43	11232	3.23	10159	4.23
		12245	2.71	10322	9.33
				10704	4.62
				11237	6.47
				12249	3.12

(B) for mass spectra obtained from Bottle 3,4, and the reference spectrum

mass spectrum from Bottle 3		mass spectrum from Bottle 4		reference spectrum of 10^5 *S.aureus* cells	
m/z	I_N	*m/z*	I_N	*m/z*	I_N
3444	15.62	3444	9.8	3445	19.69
3510	5.75	3510	13.03	3511	7.98
3570	3.94	3571	11.34	3641	6.4
3640	2.5	3640	3.44	3769	4.3
3768	2.21	3768	21.14	3879	2.51
3878	1.52	3878	7.47	3983	6.18
3982	2.31	3913	5.85	4110	3.59
4110	1.96	3983	5.78	4194	4.41
4192	1.62	4111	3.56	4308	20.4
4308	4.57	4193	8.29	4443	2.1
4441	1.75	4307	8.67	4591	1.62
4592	1.3	4444	3.05	4817	4.97
4762	4.69	4592	1.32	4879	2.23
4817	3.85	4713	2.99	5035	33.35
4877	1.21	4815	3.14	5171	2.57
5033	23.08	4878	4.75	5307	8.86
5169	1.73	5034	21.36	5387	1.62
5305	2.34	5170	1.29	5445	4.34
5385	1.26	5306	2.57	5528	57.72
5443	3.44	5386	1.02	5665	4.67
5527	59.55	5443	2.2	5820	1.22
5663	5.65	5527	36.98	5936	3.2
5822	0.98	5663	3.52	6013	1.52
6010	1.21	5819	0.41	6356	6.21
6423	2.72	5935	0.61	6425	3.41
6572	6.33	6011	0.85	6572	6.21
6702	4.46	6244	1.61	6703	3.35
6818	7.61	6355	0.67	6821	12.73
6890	100	6425	2.01	6891	100
7029	13.07	6574	3.56	7032	16.79
7165	2.11	6703	2.32	7168	1.69
7571	1.3	6819	9.14	7574	3.38
8212	1.35	6890	100	8215	2.51
8895	0.83	7029	13.67	8898	1.42
9185	0.36	7165	1.77	9185	1.16
9625	8.63	7571	0.78	9626	9.81
		8213	1.2		
		8897	0.87		
		9182	0.37		
		9627	6.3		

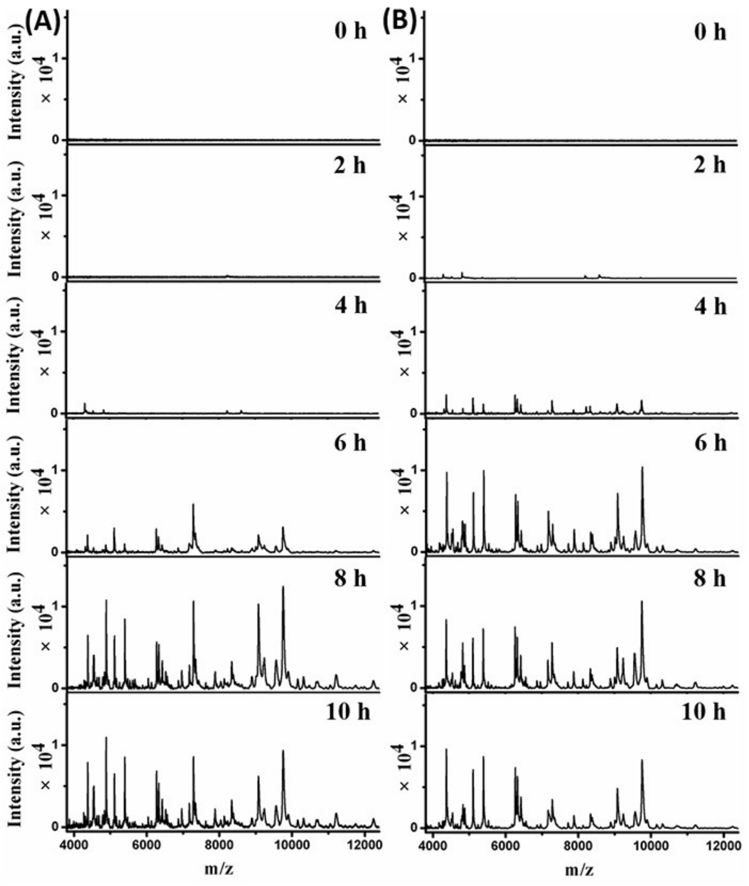

Fig.S7 Immunoaffinty MALDI-TOF mass spectra obtained from BC bottles (bottles II) with initial *E.coli* concentration of (A) 10^2 cells mL^{-1}, or (B) 10^3 cells mL^{-1} in 5 mL blood after different BC time: 0h, 2h, 4h, 6h, 8h,10h.

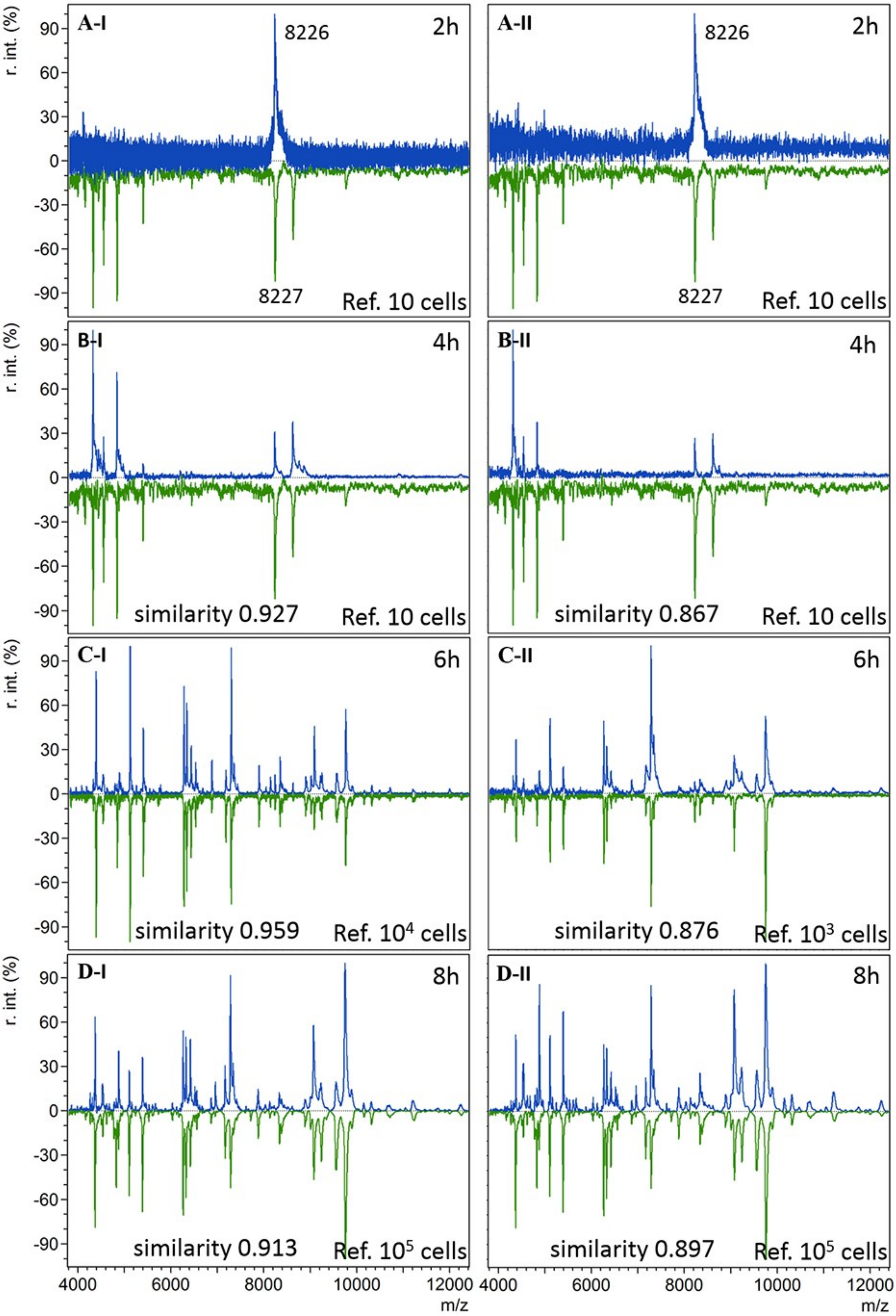

Fig.S8 Comparison between the reference spectra of *E.coli* (in green) and immunoaffinity MALDI-TOF mass spectra obtained from two BC bottles (I, II) with initial *E.coli* concentration of 10^2 cells mL^{-1} in 5 mL blood after different BC time (in blue, from Fig.4A and Fig.S7-A). I, II refer to bottle I and II, respectively. Similarity scores were calculated by the cosine correlation method. r.int: relative intensity.

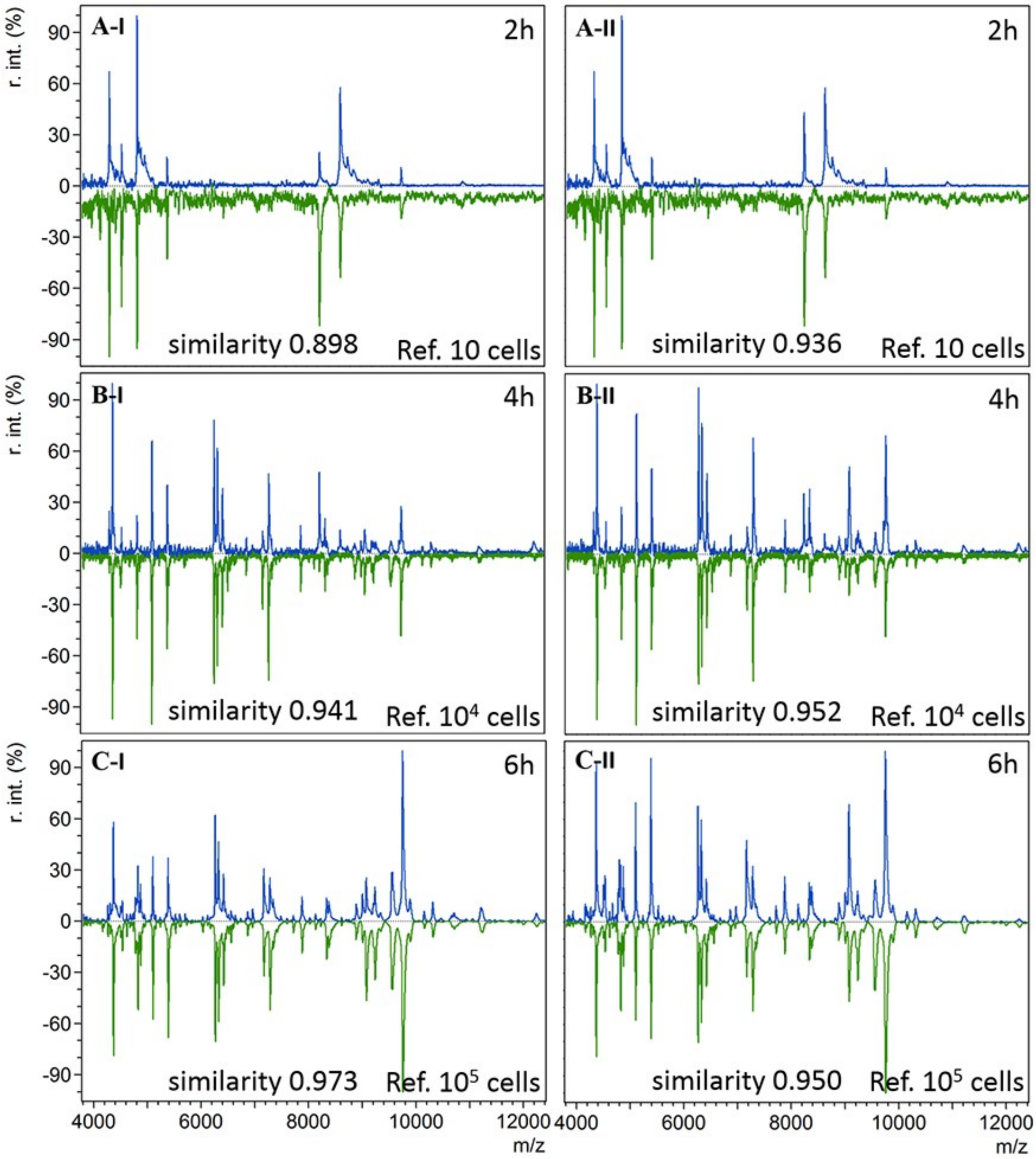

Fig.S9 Comparison between the reference spectra of *E.coli* (in green) and immunoaffinity MALDI-TOF mass spectra obtained from two blood culture bottles (I, II) with initial *E.coli* concentration of 10^3 cells mL^{-1} in 5 mL blood after different BC time (in blue, from Fig.4B and Fig.S7-B). I, II refer to bottle I and II, respectively. Similarity scores were calculated by the cosine correlation method. r.int: relative intensity.

Table S3 List of pattern matching results (with highest similarity scores) for all samples analyzed in this work by the immunoaffinity MALDI-TOF MS method. Sample name with 1-3 means three repetitions.

No.	Sample Name	Reference Spectrum	Similarity Score
1	10^8 *E.coli* cells mL^{-1} in blood serum-1	10^5 *E.coli* cells	0.926
2	10^8 *E.coli* cells mL^{-1} in blood serum-2	10^5 *E.coli* cells	0.976
3	10^8 *E.coli* cells mL^{-1} in blood serum-3	10^5 *E.coli* cells	0.956
4	10^8 *E.coli* cells mL^{-1} in whole blood-1	10^5 *E.coli* cells	0.974
5	10^8 *E.coli* cells mL^{-1} in whole blood-2	10^5 *E.coli* cells	0.981
6	10^8 *E.coli* cells mL^{-1} in whole blood-3	10^5 *E.coli* cells	0.943
7	10^8 *B.subtilis* cells mL^{-1} in blood serum-1	10^5 *B.subtilis* cells	0.986
8	10^8 *B.subtilis* cells mL^{-1} in blood serum-2	10^5 *B.subtilis* cells	0.972
9	10^8 *B.subtilis* cells mL^{-1} in blood serum-3	10^5 *B.subtilis* cells	0.970
10	10^8 *B.subtilis* cells mL^{-1} in whole blood-1	10^5 *B.subtilis* cells	0.944
11	10^8 *B.subtilis* cells mL^{-1} in whole blood-2	10^5 *B.subtilis* cells	0.979
12	10^8 *B.subtilis* cells mL^{-1} in whole blood-3	10^5 *B.subtilis* cells	0.951
13	10^8 *S.aureus* cells mL^{-1} in blood serum-1	10^5 *S.aureus* cells	0.998
14	10^8 *S.aureus* cells mL^{-1} in blood serum-2	10^5 *S.aureus* cells	0.993
15	10^8 *S.aureus* cells mL^{-1} in blood serum-3	10^5 *S.aureus* cells	0.988
16	10^8 *S.aureus* cells mL^{-1} in whole blood-1	10^5 *S.aureus* cells	0.992
17	10^8 *S.aureus* cells mL^{-1} in whole blood-2	10^5 *S.aureus* cells	0.986
18	10^8 *S.aureus* cells mL^{-1} in whole blood-3	10^5 *S.aureus* cells	0.980
19	500 *E.coli* cells mL^{-1} in blood serum-1	10 *E.coli* cells	0.975
20	500 *E.coli* cells mL^{-1} in blood serum-2	10 *E.coli* cells	0.954
21	500 *E.coli* cells mL^{-1} in blood serum-3	10 *E.coli* cells	0.980
22	8000 *E.coli* cells mL^{-1} in whole blood-1	10 *E.coli* cells	0.988
23	8000 *E.coli* cells mL^{-1} in whole blood-2	10 *E.coli* cells	0.971
24	8000 *E.coli* cells mL^{-1} in whole blood-3	10 *E.coli* cells	0.957
25	500 *B.subtilis* cells mL^{-1} in blood serum-1	10 *B.subtilis* cells	0.973
26	500 *B.subtilis* cells mL^{-1} in blood serum-2	10 *B.subtilis* cells	0.978
27	500 *B.subtilis* cells mL^{-1} in blood serum-3	10 *B.subtilis* cells	0.959
28	8000 *B.subtilis* cells mL^{-1} in whole blood-1	10 *B.subtilis* cells	0.975
29	8000 *B.subtilis* cells mL^{-1} in whole blood-2	10 *B.subtilis* cells	0.956
30	8000 *B.subtilis* cells mL^{-1} in whole blood-3	10 *B.subtilis* cells	0.954
31	500 *S.aureus* cells mL^{-1} in blood serum-1	10 *S.aureus* cells	0.993
32	500 *S.aureus* cells mL^{-1} in blood serum-2	10 *S.aureus* cells	0.984
33	500 *S.aureus* cells mL^{-1} in blood serum-3	10 *S.aureus* cells	0.979
34	8000 *S.aureus* cells mL^{-1} in whole blood-1	10 *S.aureus* cells	0.973
35	8000 *S.aureus* cells mL^{-1} in whole blood-2	10 *S.aureus* cells	0.963
36	8000 *S.aureus* cells mL^{-1} in whole blood-3	10 *S.aureus* cells	0.950
37	*S.aureus*-spiked accuracy test sample-1	10^5 *S.aureus* cells	0.991
38	*S.aureus*-spiked accuracy test sample-2	10^5 *S.aureus* cells	0.990

39	*S.aureus*-spiked accuracy test sample-3	10^4 *S.aureus* cells	0.997
40	*S.aureus*-spiked accuracy test sample-4	10^4 *S.aureus* cells	0.992
41	*S.aureus*-spiked accuracy test sample-5	10^4 *S.aureus* cells	0.991
42	*S.aureus*-spiked accuracy test sample-6	10^4 *S.aureus* cells	0.989
43	*S.aureus*-spiked accuracy test sample-7	10^4 *S.aureus* cells	0.994
44	*S.aureus*-spiked accuracy test sample-8	10^4 *S.aureus* cells	0.996
45	*S.aureus*-spiked accuracy test sample-9	10^3 *S.aureus* cells	0.994
46	*S.aureus*-spiked accuracy test sample-10	10^3 *S.aureus* cells	0.993
47	*S.aureus*-spiked accuracy test sample-11	10^3 *S.aureus* cells	0.993
48	*S.aureus*-spiked accuracy test sample-12	10^3 *S.aureus* cells	0.994
49	*S.aureus*-spiked accuracy test sample-13	10^3 *S.aureus* cells	0.987
50	*S.aureus*-spiked accuracy test sample-14	10^2 *S.aureus* cells	0.985
51	*S.aureus*-spiked accuracy test sample-15	10^2 *S.aureus* cells	0.983
52	*S.aureus*-spiked accuracy test sample-16	10^2 *S.aureus* cells	0.949
53	*S.aureus*-spiked accuracy test sample-17	10 *S.aureus* cells	0.989
54	*S.aureus*-spiked accuracy test sample-18	10 *S.aureus* cells	0.982
55	*S.aureus*-spiked accuracy test sample-19	10 *S.aureus* cells	0.978
56	*S.aureus*-spiked accuracy test sample-20	10 *S.aureus* cells	0.714
57	3 bacteria-spiked whole blood-Group A-1	10 *S.aureus* cells	0.990
58	3 bacteria-spiked whole blood-Group A-2	10^2 *S.aureus* cells	0.993
59	3 bacteria-spiked whole blood-Group A-3	10^2 *S.aureus* cells	0.991
60	3 bacteria-spiked whole blood-Group B-1	10 *S.aureus* cells	0.991
61	3 bacteria-spiked whole blood-Group B-2	10 *S.aureus* cells	0.985
62	3 bacteria-spiked whole blood-Group B-3	10 *S.aureus* cells	0.992
63	3 bacteria-spiked whole blood-Group C-1	10^2 *S.aureus* cells	0.969
64	3 bacteria-spiked whole blood-Group C-2	10 *S.aureus* cells	0.982
65	3 bacteria-spiked whole blood-Group C-3	10 *S.aureus* cells	0.970
66	Positive BC bottle 1	10^5 *E.coli* cells	0.835
67	Positive BC bottle 2	10^5 *E.coli* cells	0.888
68	Positive BC bottle 3	10^5 *S.aureus* cells	0.981
69	Positive BC bottle 4	10^5 *S.aureus* cells	0.954
70	100 cells mL^{-1} *E.coli*-BC bottle I-culure 4h	10 *E.coli* cells	0.927
71	100 cells mL^{-1} *E.coli*-BC bottle I-culure 6h	10^4 *E.coli* cells	0.959
72	100 cells mL^{-1} *E.coli*-BC bottle I-culure 8h	10^5 *E.coli* cells	0.913
73	100 cells mL^{-1} *E.coli*-BC bottle II-culure 4h	10 *E.coli* cells	0.867
74	100 cells mL^{-1} *E.coli*-BC bottle II-culure 6h	10^3 *E.coli* cells	0.876
75	100 cells mL^{-1} *E.coli*-BC bottle II-culure 8h	10^5 *E.coli* cells	0.897
76	1000 cells mL^{-1} *E.coli*-BC bottle I-culure 2h	10 *E.coli* cells	0.898
77	1000 cells mL^{-1} *E.coli*-BC bottle I-culure 4h	10^4 *E.coli* cells	0.941
78	1000 cells mL^{-1} *E.coli*-BC bottle I-culure 6h	10^5 *E.coli* cells	0.973
79	1000 cells mL^{-1} *E.coli*-BC bottle II-culure 2h	10 *E.coli* cells	0.936
80	1000 cells mL^{-1} *E.coli*-BC bottle II-culure 4h	10^4 *E.coli* cells	0.952
81	1000 cells mL^{-1} *E.coli*-BC bottle II-culure 6h	10^5 *E.coli* cells	0.950
